# A randomized controlled trial comparing treatment efficacy between rapid maxillary expansion and adenotonsillectomy in pediatric obstructive sleep apnea

**DOI:** 10.1007/s11325-025-03427-8

**Published:** 2025-07-30

**Authors:** Chalermthai Aksilp, Pattaralapa Pechpongsai, Pavinee Intakorn, Chaiyapol Chaweewannakorn, Supatchai Boonpratham, Yodhathai Satravaha, Niwat Anuwongnukroh, Supakit Peanchitlertkajorn

**Affiliations:** 1 Division of Respiratory and Critical Care Medicine, Department of Pediatrics, Queen Sirikit National Institute of Child Health, Bangkok, Thailand; 2 Department of Otolaryngology, Queen Sirikit National Institute of Child Health, Bangkok, Thailand; 3 Department of Orthodontics, Faculty of Dentistry, Mahidol University, Bangkok, Thailand

**Keywords:** Pediatric obstructive sleep apnea, Rapid maxillary expansion (RME), Adenotonsillectomy, Transverse maxillary hypoplasia

## Abstract

**Purpose:**

Adenotonsillectomy (AT) is usually recommended as the first-line therapy for pediatric obstructive sleep apnea (POSA). While AT treats soft tissue obstruction, it does not address the underlying skeletal abnormalities, such as maxillary constriction. Despite growing evidence supporting RME as a treatment option for POSA, a significant research gap remains. Therefore, we conducted a randomized controlled trial to compare treatment efficacy between RME and AT.

**Methods:**

This study recruited 24 children diagnosed with POSA and presented with concurrent significant adenotonsillar hypertrophy and transverse maxillary deficiency. Participants were randomly assigned to either AT or RME for treatment. All participants underwent Type I PSG at baseline and 6 months post-treatment. Additional assessments included dental and cephalometric analyses, the pediatric sleep questionnaire (PSQ), and the OSA-18 questionnaire. Baseline and endpoint comparisons between the two treatment groups were performed.

**Results:**

The median baseline AHI for the AT and RME groups was 7.0 (5.25–9.9) and 6.85 (5.6–8.05) events/hour, respectively. There was no significant difference between treatment groups in all parameters at baseline. The comparisons between pre- and post-treatment results showed significant improvements across multiple parameters, including AHI for both AT and RME. There was no significant difference in PSG parameters (AHI, LSAT, MSAT, and REM sleep time) and cure rate between RME and AT. The post-treatment AHI for the AT and RME groups was 1.4 (0.7–1.85) and 2.3 (1.15–5.7) events/hour, respectively. However, PSQ and OSA-18 scores were significantly higher for the RME group.

**Conclusion:**

RME and AT significantly improved sleep-related respiratory parameters in patients with POSA. RME demonstrated comparable efficacy to AT in reducing AHI and improving LAST, MSAT, and REM sleep time. However, AT provided significantly better improvement in clinical symptoms and quality of life.

**Trial registration:**

The registration of this randomized controlled trial was approved on August 24th, 2023, under the registration number TCTR20230824001.

## Introduction

The prevalence of pediatric obstructive sleep apnea (POSA) is on the rise worldwide. Recent studies reported increased prevalences of approximately 9.5–11% [1]. If POSA remained untreated, patients could suffer from delayed neurocognitive development, cardiovascular diseases, metabolic diseases, poor academic performance, and attention deficit/hyperactivity disorder [2]. Adenotonsillar hypertrophy (ATH) is the leading cause of POSA. Hence, adenotonsillectomy (AT) is usually recommended as the first-line therapy [3]. The efficacy of AT in treating POSA varied considerably across studies. The success rate also depended on the definition and apnea-hypopnea index (AHI) cut point. For example, a meta-analysis reported a success rate of 51% for AHI < 1, and the rate rose to 81% when AHI < 5 was used [4]. Furthermore, a prospective longitudinal study demonstrated a progressive increase in AHI postoperatively [5]. Nonetheless, many patients still face long waiting times for surgery due to a shortage of pediatric ENT specialists [6]. While such delays in treatment could negatively impact patients, it is worth noting that 46% of patients undergoing watchful waiting demonstrated a normalization of AHI, compared to 76% of those who received AT [7]. Overall, these studies suggested that AT may not always provide an adequate resolution of POSA.

A recent systematic review and meta-analysis reported that patients with POSA demonstrated alterations in craniofacial features, including narrow and constricted maxilla, mandibular retrognathia, and hyperdivergent mandibular growth patterns [8]. Additionally, a narrow maxillary base was also found to be correlated with a higher AHI [9]. While AT treats soft tissue obstruction, it does not address underlying skeletal abnormalities such as maxillary constriction. In contrast, orthodontic therapy targeting maxillary skeletal expansion could serve as an alternative treatment for POSA, especially in patients with narrow and constricted maxilla. Emerging evidence suggested that rapid maxillary expansion (RME) could alleviate POSA [10–12]. RME is a widely utilized orthodontic treatment for transverse maxillary hypoplasia in growing patients. It is a non-invasive procedure that necessitates neither surgical intervention nor hospitalization. It expands the maxilla by separating palatal shelves at the mid-palatal suture prior to its complete ossification. Once the desired amount of maxillary expansion is achieved, the expander is left in place during the retention phase, which lasts approximately 6 months to allow for new bone formation along the suture [13]. Possible mechanisms for RME to alleviate POSA included a significant expansion of the nasomaxillary complex. RME expanded not only the mid-palatal suture but also several cranial and circummaxillary sutures [14]. This led to a significant increase in nasal cavity volume following the expansion [15, 16]. Consequently, the increased volume facilitated a greater passage of airflow and lowered the nasal resistance in both short- and long-term follow-up [15]. In addition, CBCT studies found a 32% increase in total airway volume and a 37% increase in the cross-sectional area of the nasopharynx [16, 17]. The enlargement of the pharyngeal airway was also attributed to the improvement of tongue posture post-RME [18]. 

Regarding clinical efficacy, a 2017 meta-analysis reported 73%-95% AHI reduction among patients with mild ATH or a history of AT, and a 61% reduction in those with severe ATH [10]. A more recent meta-analysis also reported an AHI reduction of 73% in ≤ 3 years, and 77% in > 3 years of follow-up [12]. The mean blood oxygen saturation (MSAT) and the lowest blood oxygen saturation (LSAT) increased significantly following RME as well [10–12]. A prospective study showed significant improvement in clinical symptoms and quality of life in patients with POSA after maxillary expansion. However, no improvement in AHI was observed, likely due to the mild severity of the baseline AHI (1.49 ± 1.32) in this study [19]. 

Despite growing evidence supporting RME as a treatment option for POSA, a significant research gap remains. Current evidence comes mostly from non-randomized controlled studies [10, 12]. To address this and provide higher-quality evidence, we conducted a randomized controlled trial (RCT) comparing the treatment efficacy of RME directly against the standard-of-care AT in patients with POSA. This study aimed to demonstrate RME’s ability to improve AHI, other polysomnographic parameters, clinical symptoms, and quality of life, particularly for those with concurrent maxillary constriction and significant ATH, in comparison to AT.

## Materials and methods

Ethical approval was obtained from the Institutional Review Board of the Faculty of Dentistry, Mahidol University (COA.No.MU-DT/PY-IRB 2022/061.2411). The trial was registered with the Thai Clinical Trials Registry under the number TCTR20230824001.

### Participants

Patients were recruited from the Pediatric Sleep Clinic at Queen Sirikit National Institute of Child Health (QSNICH), Bangkok, Thailand. All patients are of Southeast Asian descent. Written informed consents were obtained prior to study participation. The following were the inclusion and exclusion criteria:

**Inclusion criteria**.

1. Age between 4 and 10 years.
2. Presence of signs and symptoms of POSA with AHI of ≥ 1 confirmed by type I polysomnography (PSG).
3. Friedman’s classification of tonsillar hypertrophy ≥ 2 according to an ENT evaluation [20].
4. Adenoid hypertrophy was assessed using an adenoidal-nasopharyngeal (A/N) ratio ≥ 60% (indicating significant adenoidal hypertrophy) [21].
5. Narrow and constricted maxilla with or without posterior dental crossbite according to an orthodontic evaluation.
6. Body mass index (BMI) < 25 kg/m².

**Exclusion criteria**.

1. Presence of craniofacial syndromes, including oral clefts.
2. Significant maxillary (overjet < -3 mm) and mandibular hypoplasia (overjet > 6 mm) in anteroposterior dimension according to cephalometric analyses [22].
3. Medical conditions contraindicated in AT.
4. History of orthodontic treatment and/or AT.

One hundred and seventy patients were screened. Of these, 130 were excluded: 115 did not meet the inclusion criteria, and 15 had contraindicated medical conditions and significant craniofacial abnormalities. Forty patients met all eligibility criteria. Among them, 16 were excluded: 1 already underwent AT, and 15 declined to participate. Finally, a total of 24 patients participated in the study.

### Baseline assessment (T0)

All participants underwent Type I PSG (Profusion PSG5, Compumedics, Australia) at baseline. PSG recordings were interpreted in accordance with the 2023 American Academy of Sleep Medicine guidelines [23]. Additional assessments included Friedman’s classification of tonsillar hypertrophy [20], the pediatric sleep questionnaire (PSQ) [24], and the OSA-18 questionnaire [25]. The PSQ is a validated screening tool for POSA [24]. It is also utilized to monitor therapeutic efficacy following an intervention. The OSA-18 questionnaire is a validated quality of life survey designed to measure the well-being of patients with POSA. A possible total score ranges from 18 to 126, with a higher score indicating poorer quality of life [25]. Mouth breathing was assessed through parental reports.

Dental (intercanine and intermolar widths, overjet, overbite, Angle’s classification of malocclusion) and cephalometric analyses were performed to determine baseline dentofacial characteristics of the samples. Teeth were scanned using the iTero Element scanner (Align Technology, Inc., USA) and analyzed with the OrthoCAD software (Align Technology, Inc., USA). The intermolar width was measured between the central pit of the terminal molars. Cephalometric analyses were carried out using the WebCeph (AssembleCircle Corp., Seoul, Republic of Korea). All evaluations were conducted by an orthodontic clinician blinded to patient identities and treatment groups. Both baseline and endpoint measurements were taken twice, with a two-week interval. An average of the two measurements was used for analyses. Intra-rater reliability was also calculated.

### Sample size calculation

The sample size calculation was based on Marcus et al. (2013) [7]. The calculation resulted in a minimum of 10 subjects for each treatment group with 80% power to identify an AHI difference (*p*-value < 0.05) of 2 events/hour. Assuming a 20% attrition rate, twelve participants were required for each intervention group.

### Randomization and interventions

Participants were randomized into 2 treatment groups using block randomization with a block size of 4. They were randomly assigned to either AT or RME for treatment. The randomization was performed using Stata/SE Statistical Software, version 16.1 (Stata Corp, College Station, TX, USA).

### RME

**Fig. 1:**
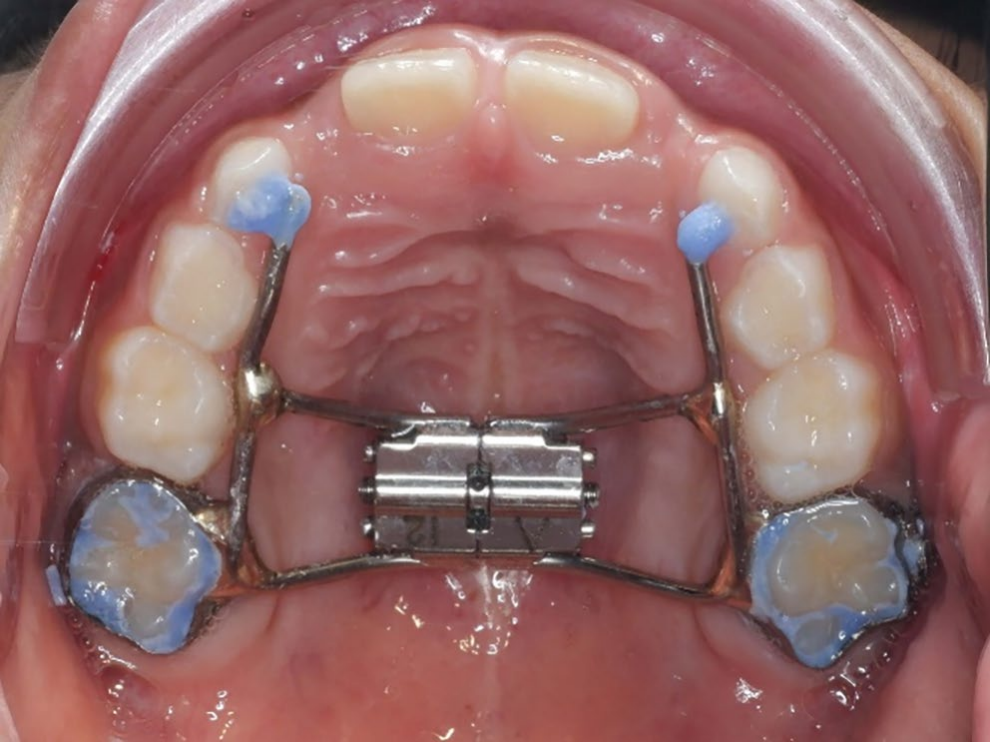
Rapid maxillary expansion (RME) device

The maxillary expansion was performed by a board-certified orthodontist at the Faculty of Dentistry, Mahidol University. A dental impression was made for RME fabrication in the initial visit. RME was then fabricated in an in-house orthodontic laboratory. It utilized either the upper first permanent molars or the upper second primary molars as anchor teeth, depending on the participant’s stage of dental development. The RME featured a mid-palatal expansion screw (Hyrax, Maxi-12, Dentaurum, Germany) with arm extensions from anchor teeth to canine. (Fig. 1) It was delivered on a subsequent visit. Parents were instructed to activate the expander twice daily for 14 days (7 mm of expansion). They also received instructions on home care and oral hygiene maintenance. The device remained in place for 6 months after achieving the desired expansion to facilitate new bone formation at the mid-palatal suture. The expander was removed following this stabilization period.

### Adenotonsillectomy

AT was performed by a board-certified ENT surgeon following a clinical practice guideline by the American Academy of Otolaryngology-Head and Neck Surgery [3] at the surgery center, QSNICH. The surgery was performed using the extracapsular technique with monopolar electrocautery. Patients remained in the hospital for 2 nights after the surgery. They were also followed up at 7 days and 1 month by the ENT surgeon.

### Endpoint assessment (T1)

**Fig. 2:**
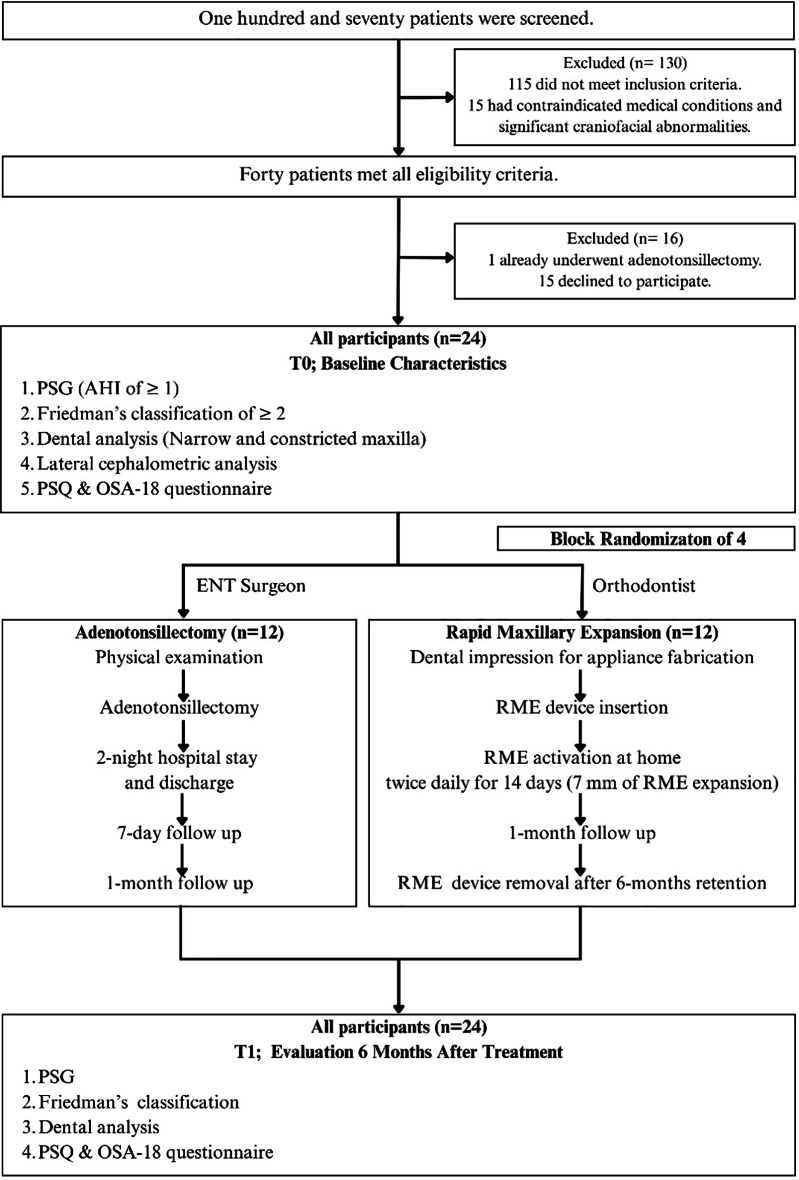
Trial flow chart. Twenty-four patients with confirmed POSA, adenotonsillar hypertrophy, and constricted maxilla, were recruited for the trial. They were assigned to either AT or RME for treatment using block randomization of 4. Baseline and 6-month assessments included PSG, Friedman’s classification, dental analysis, PSQ, and OSA-18 questionnaire

All participants underwent their assigned treatment. Follow-up assessments 6 months after treatment were performed on them. These evaluations included Type I PSG (Profusion PSG5, Compumedics, Australia), Friedman’s tonsillar hypertrophy classification [20], the PSQ [24], the OSA-18 questionnaires [25], and dental analyses (intercanine and intermolar widths). The trial flow chart is displayed in Fig. 2.

### Statistical analyses

Data distribution analysis was carried out using the Shapiro-Wilk test. For baseline and endpoint comparisons between the 2 intervention groups, independent t-tests were performed for variables that followed normal distribution (age, BMI, REM sleep time, PSQ score, OSA-18 score, overjet, overbite, intercanine width, SNA, SNB, MPA, NS-MP, MP-hyoid, facial index, and adenoidal-nasopharyngeal ratio). Mann-Whitney U tests were calculated for variables with non-normal distributions (AHI, MSAT, LSAT, intermolar width, and ANB). Categorical data (sex, Friedman’s classification, mouth breathing, Angle’s classification, and cure rate) were reported as percentages (%), and comparisons between groups were performed utilizing either the chi-square test or Fisher’s exact test.

For pre- and post-treatment comparisons within each treatment group, paired-t tests were calculated for variables with a normal distribution (REM sleep time, PSQ score, OSA-18 score, and intercanine width). Wilcoxon signed-rank test was used for parameters not following a normal distribution (AHI, MSAT, LSAT, and intermolar width). Categorical data were analyzed using the McNemar test. Intra-rater reliability was assessed using the intraclass correlation coefficient (ICC). All statistical analyses were performed using Stata/SE Statistical Software, version 16.1 (Stata Corp, College Station, TX, USA), with a significance level set at *p*-value < 0.05.

## Results

### Samples characteristics

This RCT was conducted from the end of August 2023 through December 2024. Results are presented as mean ± SD for normally distributed data or median (IQR) for non-normal distributions. Subjects had a mean age of 6.33 ± 1.71 years, with 37.5% female participants. The mean BMI was normal (16.22 ± 2.8 kg/m²). Most participants (79.2%) presented with Friedman’s classification 3. The average adenoidal-nasopharyngeal ratio (A/N ratio) was 0.78 ± 0.08, confirming significant ATH in all subjects. Most patients (87.5%) exhibited habitual mouth breathing. The median baseline AHI and LSAT were 7.0 (5.5–8.8) events/hour and 87 (83.5–87.5) %, respectively. The mean PSQ score was 0.62 ± 0.17, indicating significant clinical symptoms of POSA. The average OSA-18 score was 75.67 ± 11.88, demonstrating a moderate to severe impact on quality of life. Dental analyses revealed a narrow maxillary arch compared to established norms [26]. Angle’s Class I occlusion was observed in 66.7% of participants. Cephalometric analyses showed orthognathic maxilla, retrognathic mandible, and hyperdivergent mandibular growth pattern [22]. ICC demonstrated excellent reliability for both dental and cephalometric measurements, with values ranging from 0.97 to 0.99.

### Comparison of baseline characteristics between the two treatment groups

There was no significant difference between treatment groups (*p*-value > 0.05) observed in all parameters at baseline, confirming comparability of the pre-treatment characteristics between treatment groups. Detailed results are exhibited in Table 1.

**Table 1:** Pre-treatment (T0) comparison of baseline characteristics between AT and RME groups. Normally distributed data are presented as mean ± standard deviation (SD); non-normal ones are displayed as median with interquartile range (IQR). ° denotes degree. The following were cephalometric parameters used in this study. SNA: sagittal position of the maxilla relative to the cranial base, SNB: sagittal position of the mandible relative to the cranial base, ANB: sagittal relationship between the maxilla and mandible, MPA: mandibular plane angle, NS-MP: angle between the cranial base and mandibular plane, MP-Hyoid: linear distance from the mandibular plane lower border of the mandible to the anterosuperior point of the hyoid bone, facial index: ratio of upper anterior facial height to lower anterior facial height, A/N ratio: ratio of adenoidal distance to nasopharyngeal depth

Characteristics	All *n* = 24	AT group *n* = 12	RME group *n* = 12
Demographic data			
Age (year)	6.33 ± 1.71	6.61 ± 1.84	6.04 ± 1.61
Sex (Female %)	37.5	33.3	41.7
BMI (kg/m^2^)	16.22 ± 2.80	15.91 ± 2.94	16.53 ± 2.74
Respiratory parameters			
AHI (events/ hour)	7 (5.5–8.8)	7 (5.25–9.9)	6.85 (5.6–8.05)
MSAT (%)	98 (97–98)	97.5 (96.5–98)	98 (97.5–98)
LSAT (%)	87 (83.5–87.5)	87 (81–87)	86 (83.5–90)
Friedman’s classification			
2 (%)	16.7	8.3	25
3 (%)	79.2	83.3	75
4 (%)	4.2	8.3	0
Habits			
Mouth breathing (%)	87.5%	83.3%	91.7%
Sleep architecture			
REM sleep time (%)	7.4 ± 6.8	8.4 ± 7.9	6.4 ± 5.6
Clinical symptoms and quality of life			
PSQ (score)	0.62 ± 0.17	0.59 ± 0.16	0.64 ± 0.18
OSA-18 (score)	75.67 ± 11.88	73.5 ± 11.82	77.83 ± 12.05
Dental analysis			
Overjet (mm)	2.40 ± 2.02	2.41 ± 2.11	2.38 ± 2.02
Overbite (mm)	2.46 ± 1.88	2.58 ± 2.22	2.35 ± 1.58
Intercanine width (mm)	32.54 ± 2.59	32.03 ± 3.03	33.05 ± 2.06
Intermolar width (mm)	42.35 (40.25–46.7)	41 (40.2-46.65)	44.35 (40.95–46.7)
Angle’s classification I (%)	66.7	58.3	75
Angle’s classification II (%)	25	33.3	16.7
Angle’s classification III (%)	8.3	8.3	8.3
Lateral cephalometric analysis			
SNA (°)	81.19 ± 3.31	80.89 ± 3.02	81.48 ± 3.68
SNB (°)	77.13 ± 3.35	77.07 ± 2.83	77.19 ± 3.93
ANB (°)	4.46 (3.22–4.99)	3.92 (2.91–5.02)	4.59 (4.27–4.99)
MPA (°)	130.25 ± 5.63	129.90 ± 5.98	130.61 ± 5.50
NS-MP (°)	37.80 ± 4.70	37.69 ± 5.49	37.91 ± 4.00
MP-Hyoid (mm)	12.30 ± 3.89	11.20 ± 4.08	13.39 ± 3.52
Facial Index (%)	80 ± 7	82 ± 6	78 ± 7
A/N Ratio (%)	78 ± 8	76 ± 8	80 ± 7

### Baseline and endpoint comparison within each treatment group

The pre- and post-treatment comparisons demonstrated significant improvements across multiple parameters for both AT and RME. Both treatments significantly reduced the AHI. The LSAT increased significantly in the AT group. The REM sleep time improved significantly in the RME group. The PSQ and OSA-18 scores decreased significantly in both groups. And both treatments resulted in a significant reduction in patients with mouth breathing. Both intercanine and intermolar widths became statistically wider after treatment. Detailed results are displayed in Table 2.

**Table 2:** Post-treatment (T1) comparison within the treatment groups. Normally distributed data are presented as mean ± standard deviation (SD); non-normal ones are displayed as median with interquartile range (IQR)

Characteristics	AT		*p*-value	RME		*p*-value
	T0	T1		T0	T1	
Respiratory Parameter						
AHI (events/ hour)	7 (5.25–9.9)	1.4 (0.7–1.85)	< 0.01**	6.85 (5.6–8.05)	2.3 (1.15–5.7)	< 0.01**
MSAT (%)	97.5 (96.5–98)	98 (97–98)	0.75	98 (97.5–98)	98 (98–98)	0.37
LSAT (%)	87 (81–87)	90.5 (89-92.5)	< 0.05*	86 (83.5–90)	89.5 (86–93)	0.12
Friedman’s score (%)						
0	0	58.3	< 0.01**	0	8.3	0.46
1	0	16.7	< 0.01**	0	16.7	0.46
2	8.3	25	< 0.01**	25	25	0.46
3	83.3	0	< 0.01**	75	50	0.46
4	8.3	0	< 0.01**	0	0	0.46
Habits						
Mouth breathing (%)	83.3	8.3	< 0.01**	91.7	25	< 0.01**
Sleep architecture						
REM sleep time (%)	8.4 ± 7.9	12.3 ± 7.6	0.25	6.4 ± 5.6	12.0 ± 8.8	< 0.05*
Clinical symptoms and quality of life						
PSQ (score)	0.59 ± 0.16	0.19 ± 0.11	< 0.01**	0.64 ± 0.18	0.34 ± 0.17	< 0.01**
OSA-18 (score)	73.5 ± 11.82	38.42 ± 10.6	< 0.01**	77.83 ± 12.05	62.42 ± 9.99	< 0.01**
Dental analysis						
Intercanine width (mm)	32.03 ± 3.03	32.52 ± 2.92	< 0.01**	33.05 ± 2.06	37.18 ± 2.38	< 0.01**
Intermolar width (mm)	41 (40.2-46.65)	41.15 (40.4–46.8)	< 0.01**	44.35 (40.95–46.7)	50.75 (45.75–52.8)	< 0.01**

* Denotes statistical significance at *p*-value < 0.05

** Denotes highly statistical significance at *p*-value < 0.01

### Endpoint comparison between the treatment groups

There was no significant difference in PSG parameters (AHI, LSAT, MSAT, and REM sleep time) and the cure rate (AHI < 1) between the RME and AT groups at T1. The number of remaining patients with mouth breathing did not differ significantly between groups. However, PSQ and OSA-18 scores were significantly higher for the RPE group. The dental analysis demonstrated significantly greater intercanine and intermolar widths in the RME group. A significant tonsillar size reduction was observed in the AT group, with 58.3% of participants exhibiting complete resolution. Detailed results of comparisons between RME and AT at T1 are presented in Table 3.

**Table 3:** Post-treatment (T1) comparison between the treatment groups. Normally distributed data are presented as mean ± standard deviation (SD); non-normal ones are displayed as median with interquartile range (IQR)

Characteristics	AT	RME	*p*-value
Respiratory parameters			
AHI (events/ hour)	1.4 (0.7–1.85)	2.3 (1.15–5.7)	0.07
Cure rate (AHI < 1) (%)	41.7	16.7	0.37
Cure rate (AHI < 2) (%)	83.3	41.7	< 0.05*
MSAT (%)	98 (97–98)	98 (98–98)	0.22
LSAT (%)	90.5 (89-92.5)	89.5 (86–93)	0.64
Friedman’s score			
0 (%)	58.3	8.3	< 0.01**
1 (%)	16.7	16.7	< 0.01**
2 (%)	25	25	< 0.01**
3 (%)	0	50	< 0.01**
Habits			
Mouth breathing (%)	8.3	25	0.59
Sleep architecture			
REM sleep time (%)	12.3 ± 7.6	12.0 ± 8.8	0.93
Clinical symptoms and quality of life			
PSQ (score)	0.19 ± 0.11	0.34 ± 0.17	< 0.05*
OSA-18 (score)	38.42 ± 10.66	62.42 ± 9.99	< 0.01**
Dental analysis			
Intercanine width (mm)	32.52 ± 2.92	37.18 ± 2.38	< 0.01**
Intermolar width (mm)	41.15 (40.4–46.8)	50.75 (45.75–52.8)	< 0.01**

* Denotes statistical significance at *p*-value < 0.05

** Denotes highly statistical significance at *p*-value < 0.01

### Complication

No complications or side effects were observed in either treatment group during the trial.

## Discussion

To the best of our knowledge, this study could be the first RCT comparing therapeutic effects between RME and AT in Asian children diagnosed with OSA who presented with concurrent narrow maxilla and significant ATH. The average pre-treatment AHI demonstrated moderate severity in both groups. The dental and cephalometric analyses revealed a narrow maxilla and a retrognathic mandible with a hyperdivergent growth pattern, coinciding with results from a recent meta-analysis [8]. Comparison of baseline characteristics exhibited no significant difference between the treatment groups in all parameters, confirming comparability between them and minimizing potential biases.

This study found that both RME and AT significantly reduced AHI, the PSQ and OSA-18 scores, as well as improved mouth breathing. However, the LSAT significantly increased only in the AT group. RME also improved the LSAT, but the improvement did not reach a significant level. In contrast, a recent non-RCT prospective study conducted in Malaysian children reported a significant increase in the LSAT by 5.76% after RME [16]. The percentage of REM sleep time improved significantly only in the RME group. This finding is consistent with a meta-analysis documenting the effects of RME [11]. However, their increase was more modest, even though our samples presented with a lower REM sleep time at baseline. This suggested RME could improve sleep architecture by increasing REM sleep.

Post-treatment comparisons between the treatment groups demonstrated that RME was comparable to AT regarding the cure rate (AHI < 1), and ability to improve AHI, the LSAT, the MSAT, and the REM sleep time. These findings concur with a crossover RCT comparing protocols comprising RME and AT in reverse order [27]. The authors found no significant difference in these parameters (MSAT not reported) between AT and RME in the first phase of their RCT. Our study also found that RME reduced AHI by 66% and increased the LSAT by 4%. These amounts of improvement were comparable with meta-analyses documenting RME’s therapeutic effects on POSA [10, 12]. This study reported a comparable cure rate (AHI < 1) between RME and AT. It is worth noting that the sample size calculation was powered to detect differences in AHI as a primary outcome, while the cure rate was analyzed as a secondary outcome. Therefore, this study may have been underpowered to detect statistically significant differences for this parameter. And this finding should be interpreted with caution. When the cure rate was defined as AHI < 2, RME demonstrated a 41.7% cure rate, which was significantly lower than that of AT. The rate is comparable to the watchful waiting group (46%) in an RCT comparing AT to untreated controls [7]. Nonetheless, the watchful waiting did not demonstrate significant improvement in REM sleep duration as RME did in our study. Furthermore, patients in the watchful waiting group did not show meaningful improvement in executive function or behavior. They also experienced more complications that necessitated subsequent surgical intervention.

Overall, our results suggested that correcting maxillary base constriction could alleviate POSA at a level comparable to AT. However, AT provided significantly better improvement in clinical symptoms and quality of life. Although a recent prospective study [19] reported significant quality of life improvement following RME in patients with POSA refractory to AT, our study could be the first to identify significant improvement in clinical symptoms and quality of life in favor of AT. In addition, a recent crossover RCT demonstrated that AT was the primary treatment to significantly improve AHI, particularly in patients with more severe baseline conditions. While RME offered supplementary benefits, its impact was modest after controlling for confounding factors such as initial AHI severity, baseline LSAT, and adenotonsillar size [28]. Both interventions in our study demonstrated a similar decrease in self-reported mouth breathing. This finding confirms RME’s role in mouth breathing treatment as previously reported [29]. Although intercanine and intermolar widths became statistically wider after treatment, the increases in the AT group were clinically negligible. The increase observed in the AT group could derive from innate transverse maxillary growth of the participants.

While AT provides immediate symptomatic improvement with the removal of soft tissue obstruction, RME corrects underlying skeletal problems by promoting transverse maxillary development. RME could offer a progressive developmental advantage for a growing child’s nasomaxillary complex beyond this study endpoint assessment and possibly reduce the risk of OSA recurrence. A prospective study reported that polysomnographic changes remained stable for more than 12 years after RME [30]. Furthermore, a meta-analytic evidence demonstrated additional improvement of sleep-related respiratory parameters with longer follow-up periods [10, 12]. Considering our study’s endpoint assessment was only 6 months after treatment, an extended observation period could show a similar improvement. On the contrary, several studies reported ATH regrowth after the surgery. A retrospective study found adenoid regrowth in 13.3% of patients one year after surgery [31]. Another prospective longitudinal study documented worsening of POSA symptoms in 68% of patients after 3 years despite significant AHI improvement after the surgery [5]. Additionally, there are surgical and general anesthesia risks associated with AT [3]. The higher risk profile of AT relative to its efficacy could position RME as a preferred treatment for POSA, particularly for patients with contraindications to AT. These fundamental differences could potentially make RME part of a comprehensive approach for POSA management.

An MRI study demonstrated that adenoidal dimensions followed age-specific growth patterns, with thickness peaking at 8–9 years, depth at 4–5 years, and cross-sectional area at 6–7 years. This was followed by a gradual shrinkage of the soft tissue at the age of 10 [32]. In our study, adenoid hypertrophy was objectively assessed using the A/N ratio derived from lateral cephalometric radiographs (mean = 78 ± 8%, or a significant adenoidal obstruction in our cohort). This approach ensured that all participants were present with significant adenoid hypertrophy. It also minimized age-related variability, allowing for valid intergroup comparisons.

The strengths of our investigation included a robust experimental design with comparable baseline characteristics between groups achieved through randomization. Limitations encompassed a relatively small sample size in an Asian population and a short follow-up period. Therefore, future research should include larger and more diverse populations with longer follow-up periods. We also acknowledge the absence of a non-treatment control group as a limitation. Nevertheless, the inclusion of an untreated control arm may pose ethical challenges, such as withholding treatment, and could subject patients to adverse health consequences of untreated POSA. A combined RME and AT approach should warrant further investigation, as it could optimize therapeutic outcomes by addressing both skeletal and soft tissue obstruction.

In conclusion, both RME and AT significantly improved sleep-related respiratory parameters in children with OSA who presented significant ATH and narrow maxilla. RME demonstrated comparable efficacy to AT in reducing AHI and improving the LAST, the MSAT, and the REM sleep time. However, AT provided significantly better improvement in self-reported clinical symptoms and quality of life.

## Data Availability

The data that supports the findings of this study are available on request from the corresponding author.
